# Specific miRNA and Gene Deregulation Characterize the Increased Angiogenic Remodeling of Thoracic Aneurysmatic Aortopathy in Marfan Syndrome

**DOI:** 10.3390/ijms21186886

**Published:** 2020-09-19

**Authors:** Federico D’Amico, Elena Doldo, Calogera Pisano, Maria Giovanna Scioli, Federica Centofanti, Giulia Proietti, Mattia Falconi, Federica Sangiuolo, Amedeo Ferlosio, Giovanni Ruvolo, Augusto Orlandi

**Affiliations:** 1 Anatomic Pathology, Department of Biomedicine and Prevention, University of Rome Tor Vergata, Via Montpellier 1, 00133 Rome, Italy; fededamico92@gmail.com (F.D.); elena.doldo@uniroma2.it (E.D.); scioli@med.uniroma2.it (M.G.S.); federica.centofanti@gmail.com (F.C.); giulia.proietti.gp@gmail.com (G.P.); ferlosio@med.uniroma2.it (A.F.); 2 Cardiac Surgery, Department of Experimental Medicine and Surgery, University of Rome Tor Vergata, 00133 Rome, Italy; lindapisano82@gmail.com (C.P.); giovanni.ruvolo@uniroma2.it (G.R.); 3 Department of Biology, University of Rome Tor Vergata, 00133 Rome, Italy; falconi@uniroma2.it; 4 Medical Genetics, Department of Biomedicine and Prevention, University of Rome Tor Vergata, 00133 Rome, Italy; sangiuolo@med.uniroma2.it

**Keywords:** Marfan syndrome, thoracic aortic aneurysm, fibrillin-1, aortic smooth muscle cells

## Abstract

Marfan syndrome (MFS) is a connective tissue disease caused by mutations in the *FBN1* gene, leading to alterations in the extracellular matrix microfibril assembly and the early formation of thoracic aorta aneurysms (TAAs). Non-genetic TAAs share many clinico-pathological aspects with MFS and deregulation of some microRNAs (miRNAs) has been demonstrated to be involved in the progression of TAA. In this study, 40 patients undergoing elective ascending aorta surgery were enrolled to compare TAA histomorphological features, miRNA profile and related target genes in order to find specific alterations that may explain the earlier and more severe clinical outcomes in MFS patients. Histomorphological, ultrastructural and in vitro studies were performed in order to compare aortic wall features of MFS and non-MFS TAA. MFS displayed greater glycosaminoglycan accumulation and loss/fragmentation of elastic fibers compared to non-MFS TAA. Immunohistochemistry revealed increased CD133^+^ angiogenic remodeling, greater MMP-2 expression, inflammation and smooth muscle cell (SMC) turnover in MFS TAA. Cultured SMCs from MFS confirmed higher turnover and α-smooth muscle actin expression compared with non-MFS TAA. Moreover, twenty-five miRNAs, including miR-26a, miR-29, miR-143 and miR-145, were found to be downregulated and only miR-632 was upregulated in MFS TAA in vivo. Bioinformatics analysis revealed that some deregulated miRNAs in MFS TAA are implicated in cell proliferation, extracellular matrix structure/function and TGFβ signaling. Finally, gene analysis showed 28 upregulated and seven downregulated genes in MFS TAA, some of them belonging to the CDH1/APC and CCNA2/TP53 signaling pathways. Specific miRNA and gene deregulation characterized the aortopathy of MFS and this was associated with increased angiogenic remodeling, likely favoring the early and more severe clinical outcomes, compared to non-MFS TAA. Our findings provide new insights concerning the pathogenetic mechanisms of MFS TAA; further investigation is needed to confirm if these newly identified specific deregulated miRNAs may represent potential therapeutic targets to counteract the rapid progression of MFS aortopathy.

## 1. Introduction

Marfan syndrome (MFS) is a connective tissue disorder caused by mutations in *FBN1* gene encoding for the extracellular matrix (ECM) glycoprotein fibrillin-1, a structural component of 10–12-nm-sized calcium-binding microfibrils [1,2]. Fibrillin-1 provides a force of structural support in elastic and non-elastic connective tissue [3]. The main feature of MFS is the early development and rapid progression of thoracic aortic aneurysms (TAAs), mainly localized at the root, compared with later TAAs in non-MFS patients [1]. Recently, it has been reported that other monogenic mutations in different genes, such as *TGFBR1/2*, *SMAD3*, *MYH11* and α-SMA, are associated with aneurysmatic aortopathy [4]. Clinical findings of MFS and *FBN1* gene mutations result in a wide phenotypic variability, but early cardiovascular pathological signs remain the most life-threatening. In particular, aortopathy in MFS patients leads to root aneurysm with a risk of dissection and rupture if left surgically untreated [5]. Fibrillin-1 alterations induce aberrant transforming growth factor-β (TGF-β) and Smad-dependent pro-fibrotic signaling, as well as increased ERK1/2-mediated synthesis of matrix metalloproteinases (MMPs) [6]. In addition, MFS aortopathy is characterized by impaired aortic contractile function and endothelial-dependent relaxation induced by enhanced parietal reactive oxygen species (ROS)-mediated oxidative stress [7]. Aneurysmatic aortic dilatation in MFS patients is characterized by structural remodeling of the wall, as previously reported [8]. Independently from the genetic phenotype, aneurysmatic aortic tissue in MFS and non-MFS patients shares common characteristics, such as elastic fiber and ECM degradation due to increased elastolysis and collagenolysis of the tunica media [9]. The latter occupies nearly 80% of the aortic wall and consists of layers of smooth muscle cells (SMCs) alternating with elastic laminae; collagen types I, III and IV; glycosaminoglycans (GAGs) and proteoglycans assembled in functional lamellar units [10,11]. Lamellar units have both tensile strength and elastic recoil properties, with composition, thickness and tension maintained across species [12,13]. The earliest recognition of tissue abnormalities underlying aortic dilation in MFS was named “cystic medial degeneration” [14], previously erroneously defined as “cystic medial necrosis” due to its microscopic lacunar appearance and the relative paucity of cells [15]. Besides extracellular matrix remodeling, medial degeneration in MFS aortopathy is characterized by SMC loss and the accumulation of GAG-rich basophilic material [16]. Although initially considered to be pathognomonic of MFS aortopathy, medial degeneration by GAG-rich basophilic material accumulation is now recognized as a non-specific process which also occurrs in other pathological conditions, including hypertension-related aortopathy [17]. Moreover, increased aortic medial alcianophilia due to GAG accumulation and diffuse intimal thickening are also documented in aging [18]. Recently, Welten et al. [9] discussed the pivotal role of some specific miRNAs in vascular remodeling and TAA development. MiRNAs are small non-coding ribonucleic acids regulating both transcriptional and post-transcriptional gene expression within the 3’ untranslated region, thus inhibiting mRNA translation or promoting its degradation [19]. A single miRNA is capable of addressing multiple mRNAs and may contain multiple miRNA binding sites [20]. Some miRNAs have been documented to influence SMC function and phenotype, angiogenesis and overall vascular response to injury [21]. More recently, investigation of deregulated miRNAs in the peripheral blood of MFS patients has been suggested as a potential prognostic tool for cardiovascular manifestations [22]. Moreover, indication criteria for preventive aortic replacement are still insufficient; in this sense, biological parameters can help to overcome the current limitations of ultrasonography and magnetic resonance measurements [22].

In order to find any morphological, ultrastructural, phenotypic and biomolecular differences between MFS and non-MFS TAAs, we analyzed and compared aortic tissue and SMCs from patients undergoing surgical procedures for TAA. These findings could be useful in the future for identifying specific biomarkers for early diagnosis and progression monitoring of the disease, as well as potential therapeutic targets to prevent or delay Marfan aortopathy.

## 2. Results

### 2.1. Aneurysmatic Aortopathy Is More Severe in MFS Patients

Demographic variables and echocardiographic parameters are summarized in Table 1 and Figure S1. The mean age of MFS and non-MFS patients was 30.5 and 46 years, respectively (*p* < 0.03). No statistical difference in terms of hypertension, smoke, diabetes, obesity, peripheral artery disease, chronic pulmonary disease or cardiovascular disease familiarity was present between the two groups. Aortic root diameter was greater in MFS compared with non-MFS patients (49.5 ± 3.5 and 42.4 ± 6.8 mm, respectively; *p* < 0.001). Genetic analysis documented splicing (41.7%) and missense (58.3%) mutations of *FBN1* in MFS patients, but no clinico-pathological differences were evident between those two genotypes (data not shown).

### 2.2. Increased Tissue Remodeling Characterizes MFS TAA Tunica Media

**Figure 1:**
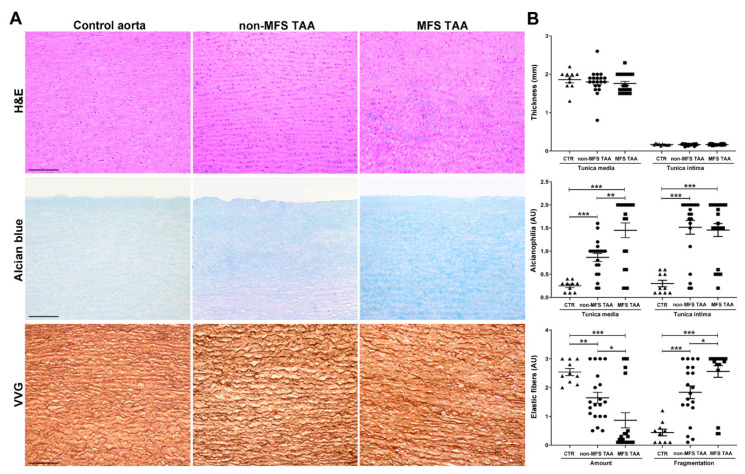
Increased alcianophilia and elastic tissue remodeling characterize Marfan syndrome (MFS) thoracic aortic aneurysm. (**A**) Representative aortic media sections stained with hematoxylin and eosin (H&E) show greater degeneration with accumulation of basophilic material in the tunica media of MFS thoracic aorta aneurysm (TAA). Alcian blue staining shows increased accumulation of alcianophilic material in MFS compared to non-MFS TAA and control aorta. Verhoeff–Van Gieson (VVG) staining documents greater loss and fragmentation of elastic fibers and a more irregular arrangement in the tunica media of MFS compared with non-MFS TAA and control aorta. Scale bars equal to 150 μm. (**B**) Bar graphs of morphometric evaluations concerning intimal and medial thickness, alcianophilia and elastic tissue loss and fragmentation in non-MFS and MFS TAA and control aortas. MFS TAA (*n* = 20), non-MFS TAA (*n* = 20) and control aorta (*n* = 10). Average are reported as means ± SEM; * *p* < 0.05; ** *p* < 0.01; *** *p* < 0.001; estimated by *t*-test. Abbreviations: AU, arbitrary units.

Microscopic examination was performed to characterize differences between MFS and non-MFS aortopathy. Foci of degeneration with accumulations of basophilic material were observed in the tunica media of both MFS and non-MFS TAA, compared with control aortas (Figure 1A, hematoxylin and eosin (H&E) staining). However, a similar intimal and medial thickness was observed in MFS and non-MFS TAAs and control aortas, despite the MFS patients’ lower mean age (Figure 1B and Figure S2). Semiquantitative evaluation of alcianophilia (Figure 1A,B, Alcian blue) documented greater GAG accumulation in the tunica media of MFS compared with non-MFS TAA (*p* < 0.01) and control aortas (*p* < 0.001). In the tunica intima, MFS and non-MFS TAA showed similar alcianophilia, which was much higher than control (*p* < 0.001). Loss and fragmentation of elastic fibers (Figure 1A,B, Verhoeff–Van Gieson (VVG) staining) were more evident in the tunica media of aneurysms compared with control aortas, and were greater in MFS than in non-MFS TAA (*p* < 0.05).

### 2.3. MMP-2 Expression and Apoptosis Are Greater in MFS TAA

**Figure 2:**
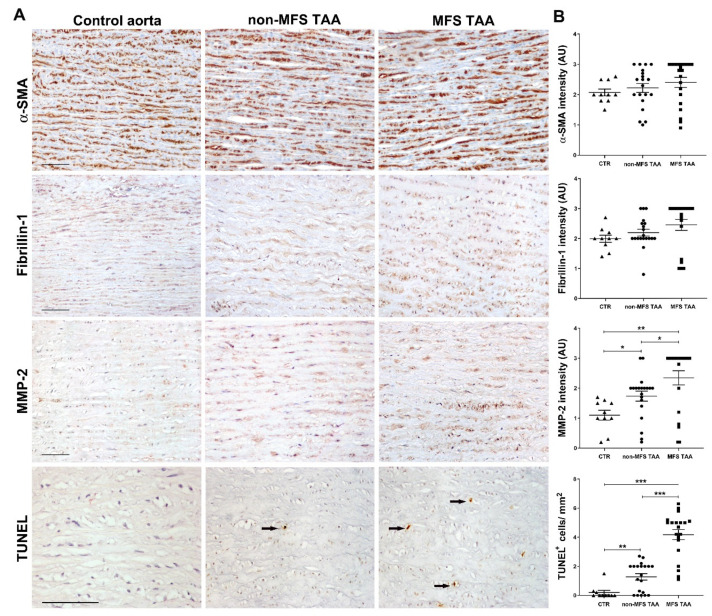
Higher MMP-2 expression and apoptosis characterize MFS thoracic aortic aneurysm. (**A**) Representative images of α-SMA, fibrillin-1, MMP-2 immunostainings and TUNEL assay in the tunica media of MFS and non-MFS thoracic aorta aneurysm (TAA) and control aorta. For TUNEL assay, only strongly positive condensed nuclei were counted (arrows). Scale bars equal to 100 μm. (**B**) Semiquantitative evaluations of immunoreactivity show increased MMP-2 expression and TUNEL^+^ cells/ mm^2^ in MFS TAA compared with non-MFS and control aorta. MFS TAA (*n* = 20), non-MFS TAA (*n* = 20) and control aorta (*n* = 10). Average are reported as means ± SEM; * *p* < 0.05; ** *p* <0.01; *** *p* < 0.001; estimated by *t*-test. Abbreviations: AU, arbitrary units.

**Figure 3:**
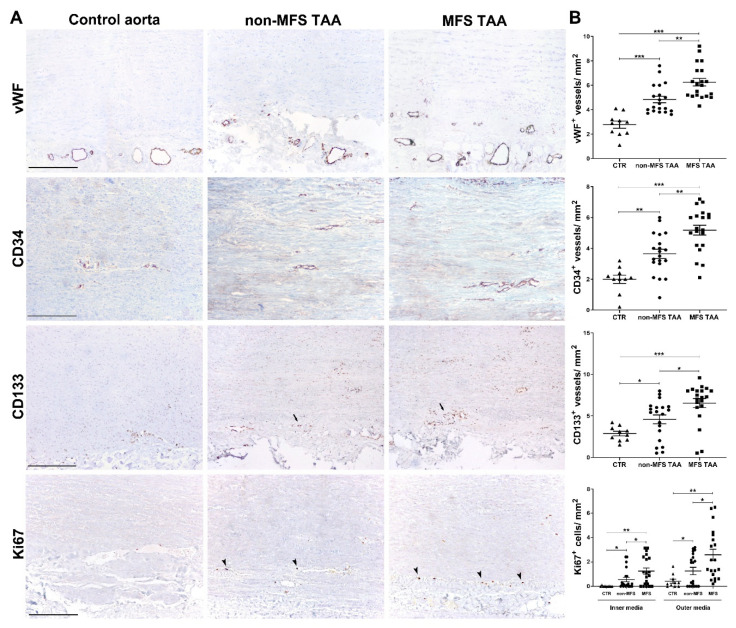
Increased angiogenesis and vascular precursor cell recruitment characterize MFS thoracic aortic aneurysm. (**A**) Representative images of vWF, CD34, CD133 and Ki67 immunostainings in the tunica media of MFS and non-MFS TAA and control aorta. Perivascular CD133^+^ cells are indicated by arrows, whereas Ki67^+^ nuclei are represented by arrowheads. Scale bars are equal to 150 μm. (**B**) Semiquantitative evaluations of immunoreactivity show increased vWF, CD34^+^ and CD133^+^ neovessels, as well as a higher number of Ki67^+^ cells/ mm^2^ mostly in the outer media of MFS TAA compared with non-MFS and control aorta. MFS TAA (*n* = 20), non-MFS TAA (*n* = 20) and control aorta (*n* = 10). Averages are reported as means ± SEM; * *p* < 0.05; ** *p* < 0.01; *** *p* < 0.001; estimated by *t*-test. Abbreviations: AU, arbitrary units.

SMC phenotype and aortic extracellular matrix were investigated by immunohistochemistry. SMCs were α-SMA-positive in MFS TAA, non-MFS TAA and control aortas (Figure 2), with a slight non-significant upward trend in MFS. Fibrillin-1 distribution appeared somewhat more uniform and extracellular in non-MFS TAA, although semiquantitative evaluation failed to find significant differences. MMP-2 expression in the tunica media was higher in MFS compared to non-MFS TAA (Figure 2B; *p* < 0.05) and lower in control aortas compared to aneurysms (*p* < 0.05 and *p* < 0.01, respectively). In-gel zymography analysis confirmed increased MMP-2 gelatinase activity in MFS compared with non-MFS TAA (Figure S3A; *p* < 0.01). Interestingly, a TUNEL assay revealed about a three-fold increase of apoptotic cells in the tunica media of MFS compared to non-MFS TAA (Figure 2; *p* < 0.001); TUNEL-positive cells were almost absent in control aortas. Double immunohistochemistry confirmed the myocyte origin of more than 90% of apoptotic cells (not shown). Nevertheless, an increased number of Ki67^+^ cells (Figure 3A,B) in the tunica media was also observed in MFS compared with non-MFS TAA (*p* < 0.001 and *p* < 0.05, respectively), suggesting an increased cell turnover in MFS TAA.

### 2.4. Increased Angiogenic Remodeling and Inflammation Characterize MFS TAA

Immunohistochemistry revealed an increased number of vWF^+^ vessels, mainly in the outer half of the tunica media, in both aneurysms compared with control aortas (Figure 3A,B; *p* < 0.001), and this was greater in MFS than in non-MFS TAA (Figure 3; *p* < 0.01). The angiogenic process was also characterized by an increase of CD34^+^ and CD133^+^ neovessels in MFS compared with non-MFS TAA (*p* < 0.01 and *p* < 0.05, respectively). Moreover, in the outer media, CD133^+^ and Ki67^+^ cells were found around neovessels in greater numbers in MFS than in non-MFS TAA (Figure S3C and Figure 3A,B; *p* < 0.01 and *p* < 0.05, respectively). Finally, we found an increase of CD4^+^ lymphocytes and CD68^+^ monocyte-macrophages mainly in the outer tunica media and adventitia of MFS compared to non-MFS TAA (Figure S3D; *p* < 0.05) and control aortas (*p* < 0.001). CD8^+^ lymphocytes were rare, with no significant differences among samples. Altogether, these findings strongly support an increased and likely early aortic remodeling in MFS TAA through angiogenesis and vascular precursor recruitment, inflammation and MMP-2 activity, as also demonstrated by the increased parietal cellularity in MFS compared with non-MFS TAA (Figure S3B).

### 2.5. Increased ECM Degeneration and SMC Morphological Changes Characterize MFS TAA

**Figure 4:**
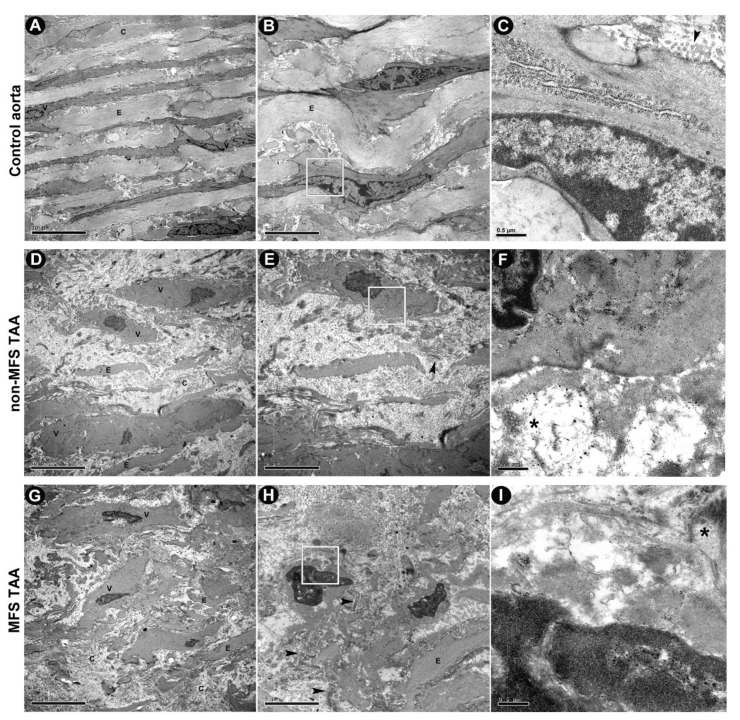
Ultrastructural investigation displays more severe extracellular matrix (ECM) degeneration and smooth muscle cell (SMC) morphological changes in MFS TAA. (**A**) Representative ultrastructural images by TEM show regular SMCs (V) and dense elastin fibers (E) and homogeneous extracellular matrix distribution in the tunica media of control aorta, with normal collagen fibers (C) either extended or twisted in longitudinal sections. (**B**,**C**) At higher magnification, regularly arranged cytoplasmic microfilaments and a more typical nuclear shape of SMCs are shown. Edematous and loose ECM, irregular fragments and damaged elastin fibers are evident in tunica media of both non-MFS (**D**–**F**) and MFS TAA (**G**–**I**). However, the latter shows a worse degenerative pattern of the tunica media, with more electron-dense sparse bundles of disorganized collagen fibrils (arrowheads), as well as thin fibrils sometimes in clusters (*). (**A**,**D**,**G)** = scale bars equal to 10 μm; (**B**,**E**,**H**) = scale bars equal to 5 μm; (**C**,**F**,**I**) = scale bars equal to 0.5 μm.

We performed an ultrastructural investigation to better characterize tissue remodeling in MFS TAA. TEM images (Figure 4A–C) documented thick and regularly arranged elastin fibers, with collagen fibers being either normal or twisted in longitudinal sections in the tunica media of control aorta. Ultrastructural examination also revealed an overall more evident edema of the tunica media ECM with thinned and more fragmented elastic fibers in both non-MFS (Figure 4D–F) and MFS TAA (Figure 4G–I) compared with control aortas. However, extracellular electron-dense sparse bundles of non-banded collagen fibers and clusters of microfibrils, as well as amorphous agglomerations of ECM, were more evident in MFS TAA. SMCs in MFS TAA appeared irregular in shape, with nuclear chromatin margination into sharply delineated huge masses. In addition, these cells appeared sometimes to be enveloped in a sort of “amorphous shell” from ECM material accumulation, as described in other pathological conditions, including age-related aortopathy [17].

### 2.6. Increased α-SMA Expression Characterizes MFS-Derived Human Aortic Smooth Muscle Cells (HASMCs) but Not Human Adventitial Fibroblasts (HAFs)

We investigated aortic SMC phenotypic differences in vitro. As shown in Figure S4A, cultured HASMCs from non-MFS and MFS TAA exhibited a similar prevalent elongated and spindle-shaped appearance and only a focal “cobblestone” morphology, considered an epithelioid synthetic phenotype [23].

Immunofluorescence demonstrated a diffuse intracytoplasmic fibrillin-1 expression in cultured HASMC from non-MFS and MFS TAA that also expressed α-SMA (Figure S4A). Blots and densitometry blot analysis documented that α-SMA levels were nearly two-fold higher in MFS compared to non-MFS TAA-derived HASMCs (Figure S4B; *p* < 0.001). HAFs showed a lower α-SMA expression than HASMCs, with no significant differences between non-MFS and MFS (Figure S4B). Blots did not document differences in fibrillin-1 expression, comparing MFS and non-MFS TAA-derived cultured HASMCs and HAFs (Figure S4C).

In vitro, HASMCs from the tunica media in the presence of serum showed a marked increase in the proliferation rate, compared to the apoptotic rate, especially in MFS (Figure S5).

### 2.7. Specific miRNA Deregulation and Gene Expression in MFS TAA

**Figure 5:**
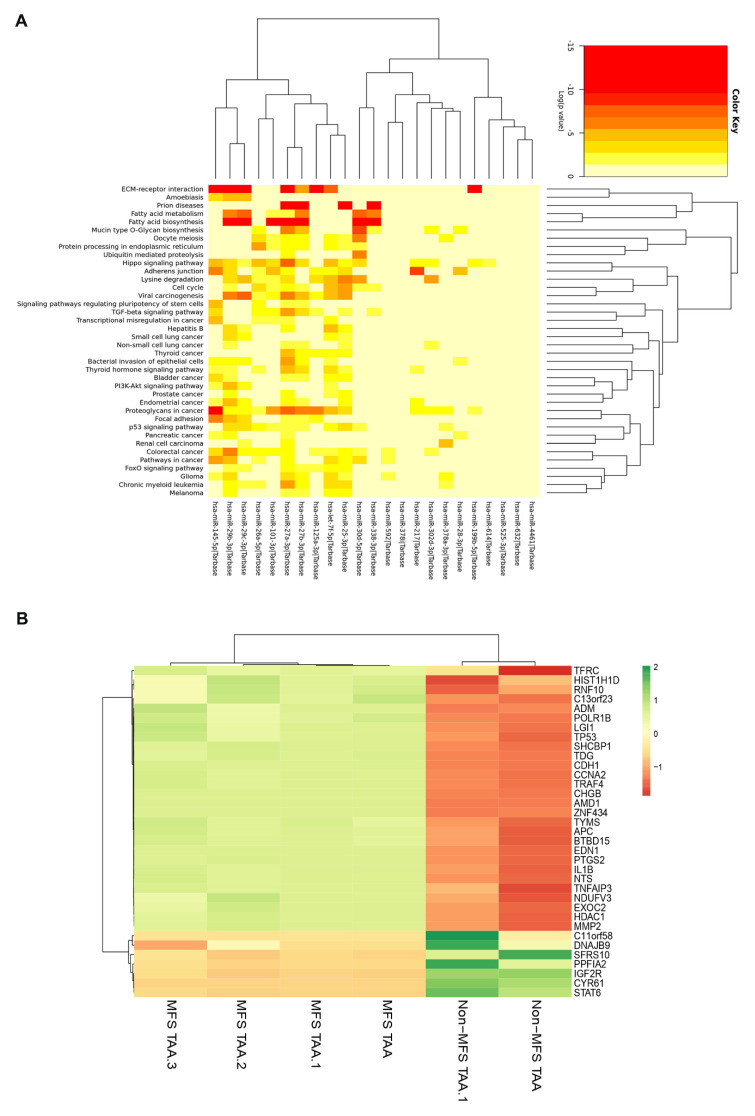
Representative intersection between deregulated miRNAs and their putative cellular pathways and expression analysis of miRNA target-genes in MFS TAA. (**A**) Heat map showing putative cellular pathways related to the deregulated miRNAs in MFS TAA medial tissue (MFS TAA, *n* = 4; non-MFS TAA, *n* = 4). Each column represents a single miRNA. (**B**) Heat map shows differential gene expression in MFS TAA medial tissue (*n* = 4) compared with non-MFS TAA (*n* = 2). Red indicates downregulation and green indicates upregulation of genes. Black suggests no significant changes in gene expression levels.

In order to evaluate if there was a specific miRNA deregulation characterizing MFS TAA in vivo, the miRNA profile of the two groups was analyzed using NanoString nCounter Technology. As reported in Supplemental Table S1, we found 25 miRNAs to be downregulated and one (*miR-632*) to be upregulated in the tunica media of MFS compared with non-MFS TAA. In particular, a heatmap analysis (Figure 5A) revealed that some of these deregulated miRNAs, like *hsa-let-7f-5p*, *hsa-miR-101-3p*, *hsa-miR-125a-3p*, *hsa-miR-199b-5p*, *hsa-miR-217*, *hsa-miR-25-3p*, *hsa-miR-27a-3p*, *hsa-miR-27b-3p*, *hsa-miR-28-3p*, *hsa-miR-29c-3p*, *hsa-miR-302d-3p*, *hsa-miR-30d-5p*, *hsa-miR-338-3p*, *hsa-miR-378a-3p*, *hsa-miR-378i*, *hsa-miR-4461*, *hsa-miR-525-3p*, *hsa-miR-592*, *hsa-miR-614*, *hsa-miR-26a-5p*, *hsa-miR-29b-3p*, *hsa-miR-145-5p* and *hsa-miR-632*, are mainly involved in the following pathways: fatty acid metabolism and biosynthesis, mucin type O-Glycan biosynthesis, protein processing in the endoplasmic reticulum, TGF-beta signaling, ECM-receptor interaction, Hippo signaling, adherens junctions, focal adhesion, p53 signaling and cell cycles, as reported in the Kyoto Encyclopedia of Genes and Genomes (KEGG).

Gene expression analysis, performed on some putative miRNA-target genes (Table S2), was carried out by means of an nCounter XT Customer Assay of gene expression. We identified 35 genes thart were differently expressed in MFS compared to non-MFS TAA (Figure 5B, Table S3 and Figure S6). Among those genes, 28 were upregulated in MFS TAA: *ADM* and *SHCBP1* (cell proliferation); *AMD1*; *APC*; *CHG B*; *CDH1* and *MMP2* (angiogenesis, adhesion, vascular and ECM remodeling); *NTS* (fat metabolism regulation); *CCNA2*; *TDG*; *TP53*; *TRAF4* and *TYMS* (regulation of cell cycle and apoptosis); *PTGS2* (inflammation and mitogenesis); *C11orf23*; *TDG*; *BTBD15*; *ZNF434* and *POLR1B* (transcriptional regulation) (KEGG database). Seven genes were downregulated in MFS TAA: *C11orf58* (chromosome 11 open reading frame 58); *CYR61* (differentiation, angiogenesis, apoptosis, and ECM formation); *DNAJB9* (anti-apoptotic); *IGF2R* (intracellular trafficking and activation of TGF-β); *PPFIA2* (disassembly of focal adhesions); *SFRS10* (mRNA processing) and *STAT6* (DNA binding transcription factor activity) (KEGG database). Overall, gene analysis strongly suggested that MFS TAA is characterized by specific miRNA and gene alterations, involving cell homeostasis, proliferation, differentiation, ECM formation and adhesion.

### 2.8. Target Prediction

A bioinformatic analysis was performed to combine the miRNAs and target genes deregulated in MFS TAA (using the DIANA-mirPath v.3 tool). We found 11 miRNAs (*hsa-miR-217*, *hsa-miR-26a-5p*, *hsa-miR-29b-3p*, *hsa-miR-25-3p*, *hsa-miR-145-5p*, *hsa-miR-27b-3p*, *hsa-miR-let-7f-5p*, *hsa-miR-30d-5p*, *hsa-miR-27a-3p*, *hsa-29c-3p* and *hsa-miR-199b-5p*), as potential regulators of seven genes (*CDH1*, *CCNA2*, *TP53*, *APC*, *TYMS*, *MMP2* and *EDN1*) (Table 2).

## 3. Discussion

In the present work, we documented increased angiogenic remodeling associated with a specific deregulation of miRNAs and their putative related genes/pathways in MFS compared to non-MFS TAA tunica media.

### 3.1. Severe Morphological Alterations Associated with Increased Tissue and Angiogenic Remodeling Characterize MFS TAA

Morphometric analysis revealed an early and more pronounced parietal remodeling in TAA tissue from MFS patients. Although intimal and medial thickness did not vary, accumulation of alcianophilic material, as well as fragmentation and loss of elastic tissue, increased in the tunica media of MFS TAA [16]. TEM observation confirmed more irregularities, with the fragmentation of elastic lamellae and the presence of abundant extracellular GAG-rich material. Increased ECM remodeling in MFS is associated with increased MMP-2 expression and gelatinase activity, according to the literature [6]. This finding is associated with increased angiogenic remodeling in MFS tunica media. This phenomenon is known to characterize the aortic tunica media during aneurysmatic and atherosclerotic processes [9,24]. We can hypothesize that a phenotypic switch of SMCs contributes to the remodeling of MFS aortas. However, the occurrence of an SMC phenotypic switch remains controversial. In human aortas, it is quite difficult to notice quantitative differences in the expression of myocitic or differentiation markers in vivo, and the great majority of SMCs in the tunica media retain a differentiative state even in pathological conditions. Kessler et al. [24] used blotting and immunostaining to demonstrate no relevant differences in myosin isoform expression of aortic SMCs from MFS and healthy patients, suggesting the absence of a marked differentiation in myofibroblasts in vivo. Similarly, routine immunostaining for vimentin and desmin of tissue sections has been performed, and did not reveal significant differences between MFS and non-MFS aortas in vivo (unpublished results). However, phenotypic synthetic switching of SMCs from non-MFS and MFS TAA has been reported in several studies [25,26,27]. In addition, conflicting results emerge from in vivo and in vitro studies and from different experimental conditions [26,28]. Dale et al. [28] described similar elastic fiber fragmentation/disorganization in the aortas of a mgR murine model of MFS, as well as increased levels of MMP-2 and MMP-9, typical of MFS aortopathy, but also found higher levels of contractile proteins, suggesting a premature switch to a more mature contractile phenotype compared to wild-type mice. A possible explanation may derive from the strong basal expression of differentiation markers in aortic SMCs in vivo, which could mask slight differences. More likely, as suggested by our TEM observations, only a small subpopulation of SMCs in MFS TAA showed subcellular changes suggesting a phenotypic synthetic switch, whereas other SMCs were surrounded by an “amorphous shell” due to ECM material accumulation, suggested a premature aging process and the death of medial SMCs. Those findings and in vitro results are in in accordance with the hypothesis that there is heterogeneity among aortic SMCs under pathological stimulation [11,29].

The increased angiogenic remodeling was characterized by a greater number of CD34^+^ and CD133^+^ neovessels, mainly in the outer tunica media of MFS TAA. In the latter, an increased number of CD133^+^ perivascular cells were found around neovessels, suggesting the recruitment of endothelial progenitor cells (EPCs) from circulation to sustain the angiogenic remodeling of the aorta. EPCs arrive in damaged vascular areas to participate in blood vessel formation and to differentiate in SMCs, as is well reported in experimental aneurysms, in line with increased SMC turnover in MFS TAA [30,31,32]. Moreover, CD133 expression is reported to be upregulated by TGFβ family members [33].

The enhanced neoangiogenesis in MFS TAA was sustained by the presence of a greater number of Ki67^+^ proliferating cells in the outer tunica media at areas of high vessel density. A significant increase of Ki67^+^ and apoptotic cells was also observed in the inner tunica media of MFS compared with non-MFS TAA, supporting the hypothesis of increased SMC turnover and cellular remodeling in MFS TAA, as reported in abdominal aortic tissue [34,35].

The increased number of CD4^+^ T lymphocytes and CD68^+^ macrophagic cells, mainly in the adventitial space and outer tunica media, confirms the presence of enhanced inflammation in MFS TAA [36,37]. Delivery of inflammatory-derived proteolytic products may also play a role in the alteration of elastic tissue in association with the parietal increased synthesis of MMP-2 or other proteolytic enzymes [38]. Increased chronic inflammation, matrix degradation and apoptosis of SMCs may also stimulate neovascularization and favor aneurysmatic aortopathy in MFS patients [38]. The increased number of structurally fragile neovessels in the outer media of the aneurysmatic aorta could also be responsible for its reduced mechanical resistance and enhanced remodeling due to an increased accumulation of bloodborne proteases and release of angiogenic factors [9]. As a consequence, the increased parietal cellularity in MFS compared to non-MFS TAA is likely due to the significant increase in the number of inflammatory/recruited cells and vascular precursors from inflammation and angiogenetic remodeling in MFS TAA. Thus, the higher cellularity found in MFS tunica media may reflect not only the increased SMC turnover, but also the increased angiogenic remodeling and the inflammatory recruitment that characterizes the MFS aortic wall.

### 3.2. Phenotypic Differences of SMCs from MFS TAA In Vitro

Our in vitro studies endorsed the hypothesis that the angiogenic remodeling in aneurysmatic aortopathy is also characterized by differences in SMC features [9]. HASMCs, but not HAFs, from MFS TAA showed higher α-SMA expression compared to those from non-MFS TAA. The increased expression of α-SMA is generally considered a characteristic of the myofibroblastic phenotype, at least in some human pathological conditions [39]. Furthermore, HASMCs from the tunica media in the presence of serum showed a marked increase in the proliferation rate, compared to the apoptotic rate, especially in MFS. This phenomenon is likely due to the in vitro culture conditions, which favor the switch from a differentiation state towards a more synthetic phenotype after a few passages [26]. However, the increased proliferation rate showed by MFS HASMCs, compared with those from non-MFS TAA, supports the in vivo data concerning the higher SMC turnover in MFS TAA.

### 3.3. Specific miRNA and Related Gene Deregulation Characterizes MFS Aneurysmatic Aortopathy

Increased vascular remodeling and phenotypic changes in MFS TAA are associated with specific miRNA deregulation in aortic tunica media. Recently, an interesting review by Welten et al. [9] reported that some specific miRNAs, like miR-126 and miR-155, have been found to be frequently deregulated during vascular remodeling and TAA development. Lately, the role of miRNA deregulation in MFS patients has been highlighted [22]. In particular, in order to identify earlier predictive markers of MFS TAA, miRNA deregulation in peripheral blood of MFS patients compared with healthy subjects was documented [22]. However, those blood-derived deregulated miRNAs may not specifically refer to MFS-related aortopathy, but merely to the aneurysmatic conditions.

We documented for the first time that MFS is characterized by a specific pattern of miRNA deregulation compared with non-MFS aneurysmatic aortopathy. We found 25 downregulated miRNAs and one upregulated miRNA in MFS tunica media. Bioinformatics analysis revealed that some deregulated miRNAs of MFS aortopathy are implicated in cell proliferation, growth arrest, ECM structure/function and TGF-β signaling [40]. Among these deregulated miRNAs, miR-26a was previously found to be downregulated during aortic aneurysm formation and apoptosis [41]. The downregulation of miR-29 was proven to induce the synthesis of collagen types I and III and to regulate cardiac fibrosis [42]. It has also been reported that miR-29 downregulation is likely to be responsible for the compromised aortic distensibility and systemic compliance observed in MFS aortopathy [20]. Our findings concerning the regulatory function of miRNA-29b and its potential role as a therapeutic target confirm those previously reported in studies in murine models of aortic aortopathy using *Fbn1*^C1039G/+^ Marfan mice [43,44]. It is well known that MiR-29b is an important marker of proliferation and vascular remodeling during aneurysm development [44] and plays an important role in regulating apoptosis and extracellular matrix abnormalities in the MFS aortic wall [45]. MiR-145 and miR-143 deregulation was related to structural modifications of the aortic wall due to incomplete SMC differentiation [46]. In particular, miR-143 downregulation was reported to influence the SMC phenotype in vitro and to alter contractile capacity, likely inducing loss of structural integrity of the tunica media [47]. Recently, the involvement of miR-29b, miR-26a, miR-101 and miR-27b in the negative regulation of angiogenesis by vascular endothelial growth factor (VEGF) inhibition was documented [48,49,50,51]. Moreover, miR-145 and miR-27, both downregulated in MFS, have been indicated among “the top five miRNAs” involved in the vascular remodeling process, atherosclerosis and restenosis formation, aneurysm formation and neovascularization [9].

**Figure 6:**
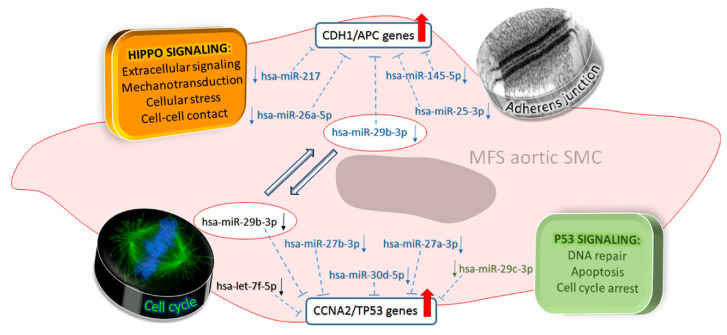
Schematic representation of miRNAs, their related gene targets and cellular pathways likely involved in MFS aortopathy. In blue, miRNAs involved with the Hippo signaling pathway (related to adherens junctions) and TP53 signaling (related to cell cycle); in black, miRNAs belonging to the “cell cycle” pathway; in green, miRNAs belonging to the “P53 signaling” pathway. MiR-26a and miR-217 are involved in Hippo signaling, whereas miR-145 and miR-25 are involved with adherens junction regulation, and their downregulation promotes the activation of CDH1/APC genes, allowing for matrix remodeling. MiR-27b, miR-30d, miR-27a and miR-29c are involved in P53 signaling, whereas let-7f-5p is involved in “cell cycle signaling”, likely through CCNA2 and TP53 upregulation. Downregulation of the miR-29 family induces the expression of collagen types I and III, influencing cardiac fibrosis, which is likely responsible for the compromised aortic distensibility and systemic compliance in MFS patients.

It is likely that the greater downregulation of those miRNAs favors the pro-angiogenic process and sustains the increased outer media angiogenesis in MFS TAA outer media. We found miR-632 to be the only upregulated miRNA in MFS, compared to non-MFS TAA. MiR-632 is an important epigenetic regulator of DNAJB6 (a member of the HSP40 family) and of the epithelial–mesenchymal transition in cancer stem cells [52]. The upregulation of miR-632 was associated with an increased expression of CD133 stem cell marker in MFS TAA. CD133 was also reported to downregulate miRNAs like miR-199b-5p and miR-145 [53], which we found to be decreased accordingly in MFS TAA. Surprisingly, gene expression analysis, despite the small sample size, documented the downregulation of DNAJB9, which belongs to the class of ER-resident DNAJs/HSP40s [54]. These data suggest that DNAJB9 could be indirectly regulated by miR-632 in vascular SMCs. Among the other deregulated genes in MFS, CYR61 is reported to be involved in cell adhesion to the ECM [55,56]. We found that CYR61 downregulation is associates with increased expression of MMP-2, EDN1 and CDH1, all of which are important mediators of ECM remodeling. In silico analysis also revealed 11 miRNAs which could be possible regulators of MMP-2, EDN1, CDH1, CCNA2 and TP53 genes in MFS TAA. In addition, we observed the upregulation of CCNA2 and TP53 genes, both involved in cell cycle control [57,58,59]. It is worthy of note that TP53 is also upregulated by TGF-β1 signaling, and strongly dysregulated in MFS tissues [60,61]. A scheme summarizing the main signaling pathways involved in MFS TAA is presented in Figure 6.

## 4. Materials and Methods

### 4.1. Sample Collection

For this study, MFS (*n* = 20) and non-MFS (*n* = 20) patients undergoing elective surgical procedures for TAA between 2018 and 2019 in the Department of Cardiosurgery of Tor Vergata University of Rome were enrolled. MFS diagnosis was made according to clinical criteria of the Ghent nosology [62]. Eligibility criteria were patients aged 20–50 years with ascending aorta aneurysm without coronary artery disease, endocarditis, aortic dissection, renal failure, liver disease or tumor, and a good ejection fraction.

MFS patients’ age ranged from 17 to 60 years (mean = 30.5 years) and non-MFS patients’ age ranged from 17 to 55 years (mean = 46 years). Diameter evaluation of ascending aorta was made both preoperatively and in the operating room by transthoracic echocardiography and transesophageal echocardiography estimations were performed as follows: estimating dimensions of aortic annulus, sinuses of Valsalva and proximal ascending aorta (above 2.5 cm of the sinotubular junction) in the parasternal long-axis view and evaluating those of the aortic arch from the suprasternal view (Table 1 and Figure S1). Echocardiography-derived sizes were reported as internal diameter size [63]. Color Doppler was used to assess the presence and severity of aortic regurgitation and stenosis. Measurements of aortic root and ascending aorta diameters were also carried out using helical computed tomography image analysis techniques [63]. Mutation analysis was performed on MFS patients by means of Ion S5 Next Generation Sequencing (Thermo Fisher Scientific, Waltham, MA, USA) to analyze mutations of the *FBN1* gene. All variant findings were then validated using Sanger Sequencing. The surgical procedure most often used was the isolated ascending aorta replacement or button Bentall operation, a modification of the original technique described by Kouchoukos et al., 1991 [64]. All operations were performed using crystalloid or hematic cardioplegia. As a control, we collected thoracic aorta tissue samples (*n* = 10) of patients who died from non-cardiovascular diseases (mean age = 49.1 years) from our autoptic archive of Anatomic Pathology. The Local Ethics Committee approved the study (protocol n.179/18–01-Aorta-2018) and written informed consent was obtained from each patient prior to the study. This study conformed to the principles outlined in the Declaration of Helsinki.

### 4.2. Microscopic, Histochemical and Immunohistochemical Analysis

Intimal and medial thickness and cellularity were evaluated on serial four-µm-thick sections of 10% neutral-buffered formalin-fixed paraffin-embedded aortic tissues stained with hematoxylin and eosin. Alcian blue and Verhoeff–Van Gieson staining were used to evaluate extracellular GAG accumulation and elastic fiber loss and fragmentation, respectively [13,18]. Immunoreactions with rabbit monoclonal anti-CD4 (1F6, 1:100), anti-CD8 (C8144B, 1:100), mouse monoclonal anti-CD68 (M0814, 1:100), anti-Ki67, anti-CD34 and anti-vWF (Von Willebrand factor) were performed using a Ventana BenchMark (Roche Diagnostics, Milan, Italy) autostainer system, as reported [65]. Serial sections were also reacted with mouse monoclonal anti-α-smooth muscle actin (α-SMA 1:100; DAKO Cytomatic, Glostrup, Denmark, Ab1A4) and anti-fibrillin-1 (1:25; Chemicon International, North Ryde, NSW, Australia, Ab53076), rabbit polyclonal anti-metalloproteinases-2 (MMP-2, 1:150; Thermo Scientific Pierce, IL, USA, Ab-7 B1537-P1) and anti-CD133 antibodies (1:20; Abcam, Cambridge, United Kingdom, Ab16518). Positive and negative controls were used [65]. Anti-fibrillin-1 immunostaining was repeated by using a mouse monoclonal antibody (1:150; Merck Millipore, DarMFStadt, Germany), which gave similar results (not shown). Histomorphometric analysis for intimal and medial thickness measurements was performed under a light microscope (Eclipse E600, Nikon, Tokyo, Japan) and images were captured using a digital camera (*DXM1200F*, Nikon, Tokyo, Japan) connected to the ACT-1 ImageJ software (NIH, Bethesda, MD, USA).

### 4.3. Apoptosis Assay In Vivo

The evaluation of apoptosis was performed on formalin-fixed paraffin-embedded aortic tissue sections by using an in situ TUNEL labeling detection kit (Roche Inc., Kingsland St, Nutley, NJ, USA), as reported [66]. Ten randomly selected high-power fields (400×) were analyzed and only strongly positive condensed nuclei were considered.

### 4.4. Zymography

Relative aortic MMP-2 gelatinase activity was examined by substrate-specific zymographic analysis as described previously [59]. The aortic extracts (10 μg total protein) were loaded onto electrophoretic gels (SDS-PAGE) containing 1 mg/mL of gelatin. After SDS-PAGE, gels were washed and incubated for 12 h in an MMP substrate buffer at 37 °C. After incubation, gels were stained using 0.1% Coomassie blue and destained in water. Densitometric analysis of gelatinase activity was performed in triplicate, measuring the intensity (uOD) for each band [67].

### 4.5. Ultrastructural Study

For transmission electron microscopy, small (1 mm) tunica media samples from TAAs were fixed in Karnovsky’s solution, processed and embedded in EPON 812, as reported [68]. Ultrathin sections were counterstained with uranyl acetate and lead citrate and photographed with a Hitachi H-7100FA microscope (Hitachi, Tokyo, Japan).

### 4.6. Cell Culture Proliferation and Apoptosis In Vitro

Human aortic smooth muscle cells (HASMCs) were isolated from the tunica media of residual TAA tissue samples and cultured as reported [11]. Human adventitial fibroblasts (HAFs) were obtained by enzymatic digestion of aortic adventitia [69]. HASMCs and HAFs were used between the 2nd and 5th passage.

For apoptosis, non-MFS and MFS HASMCs were fixed in 4% paraformaldehyde. Apoptotic cells were detected by using an in situ TUNEL labeling detection *kit* (Roche Inc., Kingsland St, Nutley, NJ, USA), as reported [67]. To evaluate proliferative activity, HASMCs were seeded at a density of 3 × 10^3^ cells/cm^2^ in 24 multi-well plastic plate and, after serum starvation, were cultured in DMEM supplemented with 10% FBS. Proliferation was expressed as the ratio between counted and seeded HASMCs [67].

### 4.7. Western Blotting

After extraction, content determination and electrophoresis on a 5–20% gradient gel, 50 μg of proteins were transferred to nitrocellulose filters (Schleicher and Schuell, Dassel, Germany) and incubated with anti-α-SMA (1:250; DAKO) and anti-fibrillin-1 (1:200; Merck Millipore, Massachusetts, USA) followed by a horseradish peroxidase (HRP)-conjugated goat anti-mouse IgG (1:5000; Sigma Aldrich, Saint Louis, MI, USA). Normalization was performed using anti-α-tubulin (Sigma Aldrich, Saint Louis, MI, USA). Detection and quantification were carried out in triplicate experiments, as reported [11].

### 4.8. Immunofluorescence

For immunofluorescence, HASMCs were fixed in methanol for 5 min at −20 °C and incubated with mouse monoclonal anti-α-SMA (1:100; DAKO Cytomatic, Glostrup, Denmark) and anti-fibrillin-1 (1:150; Merck Millipore, MA, USA) antibodies for 1 h at room temperature. Then, fluorescein isothiocyanate-labeled or rhodamine-labeled secondary antibodies (Life Technologies, Termo Fisher Scientific, MA, USA) were used [68]. Nuclei were counterstained using Hoechst 33258 (Sigma Aldrich, Saint Louis, MI, USA). HASMCs were photographed with ACT-1 camera controller software connected to a DXM1200F digital camera (Nikon).

### 4.9. MiRNA Profile and Pathway Enrichment Analysis

MiRNAs were isolated from cryopreserved non-MFS (*n* = 4) and MFS (*n* = 4) tunica media tissue samples [11] using a mirVana miRNA Isolation Kit (Termo Fisher Scientific, MA, USA) according to manufacturer’s protocols. MiRNA analysis was performed by Pharmadiagen (CBM scrl, Trieste, Italy) in triplicate. Fold change was considered significant for values greater than 2 or less than −2. MiRNAs were considered differentially expressed with a false discovery rate (FDR) < 5% (Table S1).

To assess the regulatory role of miRNAs and to identify miRNA-related pathways potentially involved in MFS aortopathy, we performed a functional characterization using DIANA TOOLS mirPath v.3 software (http://www.microrna.gr/miRPathv3) [70] according to the Kyoto Encyclopedia of Genes and Genomes (KEGG). The FDR method was implemented to select those biological pathways with a threshold of significance defined by *p* < 0.05.

### 4.10. Gene Expression and Data Analysis

After isolation with Trizol reagent (Invitrogen, Carlsbad, CA, USA), RNA samples were analyzed using the nCounter XT Customer Gene Expression Assay kit (Table S2, NanoString Technologies, Seattle, WA, USA) according to the manufacturer’s instructions. RNA samples (100 ng) were incubated in the presence of target-specific capture and reporter probes at 65 °C for 16 h. After complete hybridization, samples were loaded onto the Prep Station (NanoString Technologies, Seattle, WA, USA), according to the manufacturer’s recommendations. A Digital Analyzer (NanoString Technologies, Seattle, WA, USA) was used for imaging and analysis of the abundance of specific RNAs [71].

Data analysis was performed using nSolver Software, version 4.0. Each sample was normalized on four optimal housekeeping genes with the most stable expression for the analysis from the full custom panel, automatically selected by the software: *ACTB* (NM_001101.2), *LDHA* (NM_005566.1), *THBS1* (NM_003246.2) and *UBE2B* (NM_003337.2). Fold change was considered significant for values greater than 1.5 and less than −1.5 (Table S3).

### 4.11. Statistical Analysis

For histomorphometric analyzes, blinded measurements were performed by two independent pathologists, with an interobserver reproducibility > 95%. In vivo and in vitro results were expressed as the mean ± standard error mean (SEM), and differences were analyzed by means of Student’s *t*-test. Statistical analysis was performed using SPSS 22 software (IBM SPSS Statistics, Chicago, IL, USA) and differences were considered statistically significant for *p* < 0.05. Demographic and clinical analyses were performed with STATA 14.1 software (Stata Corp, College Station, TX, USA). The Fisher test for qualitative variables and the Kruskal–Wallis test for quantitative variables of clinical data were used. Normality and homoscedasticity were verified through the Shapiro–Wilk and Fisher tests.

## 5. Conclusions

We documented that several differentially expressed miRNAs and genes characterize MFS TAA and are associated with increased angiogenic parietal remodeling, compared to non-MFS TAA. In particular, the deregulation of specific miRNAs and their target genes could be responsible for alterations of related signaling pathways and processes related to adherens junctions, inflammation, lipid metabolism and extracellular matrix remodeling, which characterized MFS aortic wall changes. These degenerative processes may sustain the early development and rapid progression of TAA in MFS patients. Further studies are needed to confirm if deregulation of those MFS-specific miRNAs may represent possible biomarkers for the early diagnosis and the monitoring of aortopathy progression, as well as promising therapeutic targets to prevent or delay TAA development in MFS patients.

## Figures and Tables

**Table 1:** Demographic and clinical characteristics of the study population.

Variables	*n* Patients	Marfan	*n* Patients	Non Marfan	*p*-Value	
Age	20	30.5 [17–60]	20	46 [17–55]	0.025	w
BMI	20	25.1 ± 3.7	20	26.3 ± 3.4	0.316	t
BSA	20	1.9 [1.4–2.4]	20	2 [1.4–2.2]	0.704	w
Hypertension	20	5 (25%)	20	11 (55%)	0.105	f
Dyslipidemia	20	1 (5%)	20	5 (25%)	0.182	f
Diabetes (insulin-dependent)	20	1 (5%)	20	1 (5%)	1.000	f
Diabetes (not insulin-dependent)	20	0 (0%)	20	1 (5%)	1.000	f
Smoke	20	6 (30%)	20	10 (50%)	0.333	f
Obesity	20	1 (5%)	20	2 (10%)	1.000	f
Peripheral artery disease	20	3 (15%)	20	2 (10%)	1.000	f
Reoperation	20	1 (5%)	20	0 (0%)	1.000	f
Chronic Pulmonary Disease	20	0 (0%)	20	1 (5%)	1.000	f
Creatinine mg/dl	20	0.8 [0.6–1.1]	20	0.8 [0–1.3]	0.978	w
Cardiovascular Disease Familiarity		3 (15%)		10 (50%)	0.041	f
NYHA	20		20		0.537	f
*I*		15 (75%)		11 (55%)		
*II*		2 (10%)		5 (25%)		
*III*		2 (10%)		3 (15%)		
*IV*		1 (5%)		1 (5%)		
Atrial Fibrillation	20	0	19	1 (5.3%)	0.487	f
EuroScore II	20	0 [0-0.1]	20	0 [0–0.1]	0.924	w
Left Ventricle telediastolic diameter	20	56.1 ± 6.9	20	54.3 ± 7.8	0.433	t
Left Ventricle telesistolic diameter	20	37.6 ± 5.3	20	36 ± 7.1	0.408	t
Left Ventricle telediastolic volume	20	141.9 ± 28.7	20	142.2 ± 40.1	0.982	t
Left Ventricle telesistolic volume	20	56 [54–90]	20	60 [31–111]	0.849	w
Ejection Fraction %	20	60 [49–76]	20	60 [45–70]	0.838	w
Pulmonary artery systolic pressure	20	0 [0–35]	20	25 [19–44]	<0.001	w
Aortic Valve Stenosis	20	2 (10%)	20	4 (20%)	0.661	f
Aortic Valve Regurgitation	20	2 (10%)	15	13 (86.7%)	<0.001	f
Aortic valve morphology	20		20		1.000	f
*bicuspid*		14 (70%)		13 (65%)		
*tricuspid*		6 (30%)		7 (35%)		
Aortic root diameter (mm)	17	49.5 ± 3.5	20	42.4 ± 6.8	0.001	ξ
Sino-tubular Junction diameter (mm)	6	31.3 ± 17.9	3	35.7 ± 4	0.700	t
Ascending Aorta Diameter (mm)	10	29.8 ± 12.5	19	44.9 ± 7.4	0.001	t
Missense mutation	12	60%				
Splicing mutation	8	40%				

t = *t*-test, w = Wilcoxon rank sum test, ƒ = Fisher test, ξ = Welch test.

**Table 2:** List of deregulated miRNAs and their putative target genes.

miRNAs	Target Genes
hsa-miR-217 ↓	CDH1 ↑
hsa-miR-26a-5p ↓	
hsa-miR-29b-3p ↓	
hsa-miR-25-3p ↓	
hsa-miR-145-5p ↓	
hsa-miR-27b-3p ↓	CCNA2 ↑
hsa-miR-29b-3p ↓	
hsa-miR-let-7f-5p ↓	
hsa-miR-30d-5p ↓	
hsa-miR-27a-3p ↓	
hsa-miR-29b-3p ↓	TP53 ↑
hsa-miR-27b-3p ↓	
hsa-miR-let-7f-5p ↓	
hsa-miR-27a-3p ↓	
hsa-miR-29c-3p ↓	
hsa-miR-29b-3p ↓	APC ↑
hsa-miR-29c-3p ↓	
hsa-miR-29b-3p ↓	MMP2 ↑
hsa-miR-145-5p ↓	
hsa-miR-29c-3p ↓	
hsa-miR-199b-5p ↓	
hsa-miR-let-7f-5p ↓	EDN1 ↑
hsa-miR-29b-3p ↓	TYMS ↑

↑: upregulated; ↓: downregulated.

## Supplementary Materials

Supplementary materials can be found at https://www.mdpi.com/1422-0067/21/18/6886/s1.
