# Supplementary material for: Specific miRNA and Gene Deregulation Characterize the Increased Angiogenic Remodeling of Thoracic Aneurysmatic Aortopathy in Marfan Syndrome
Source: Int J Mol Sci. 2020 Sep 19;21(18):6886. doi: 10.3390/ijms21186886 (PMC7555983; doi:10.3390/ijms21186886)
Supplement: Supplementary file 1 [file ijms-21-06886-s001.pdf]

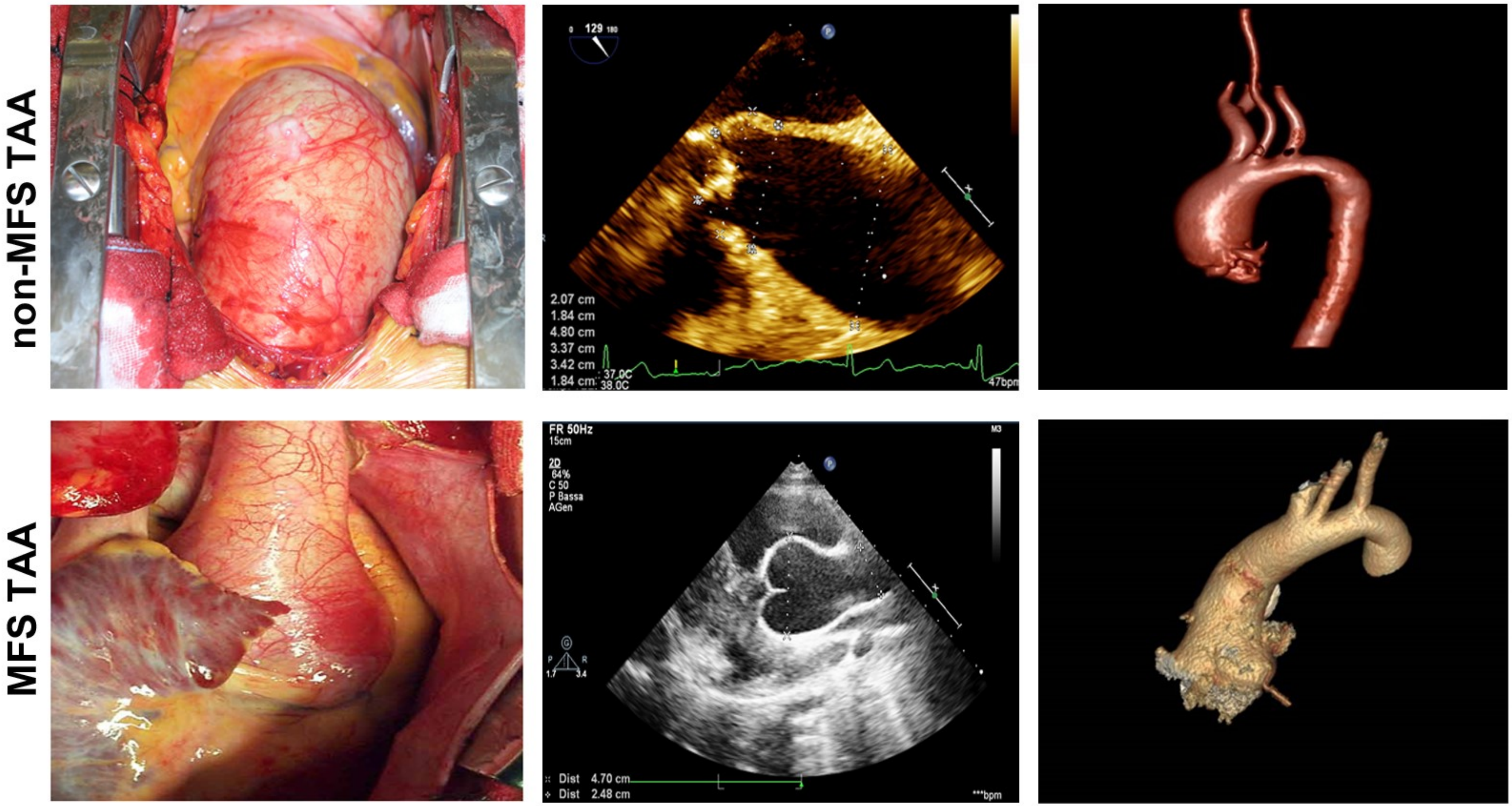

Supplemental Figure I

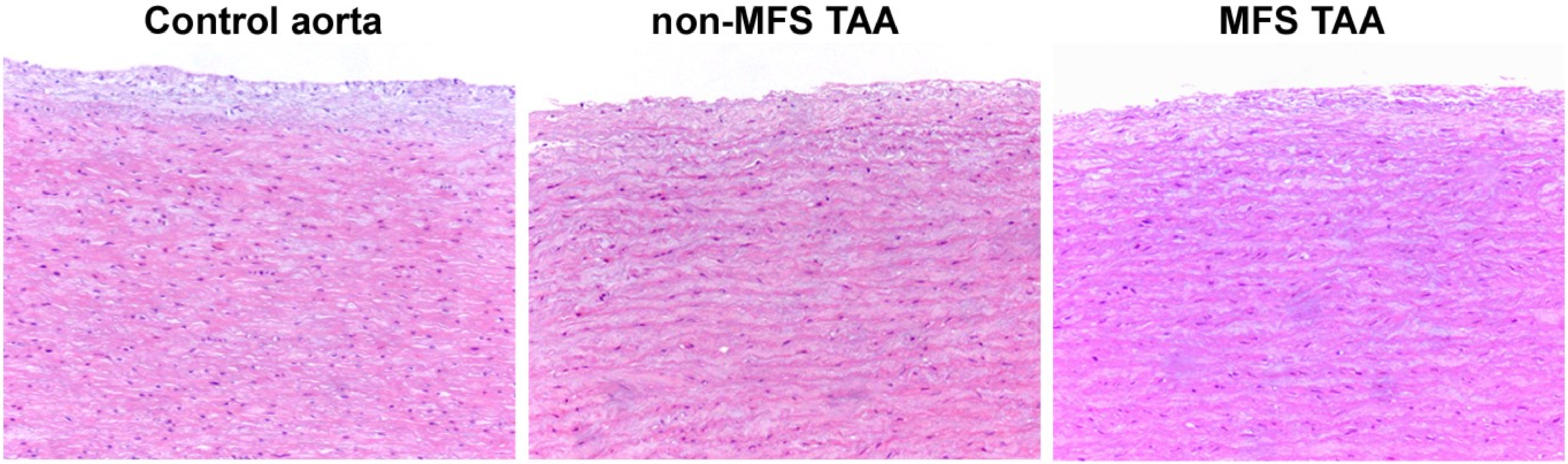

Supplemental Figure II

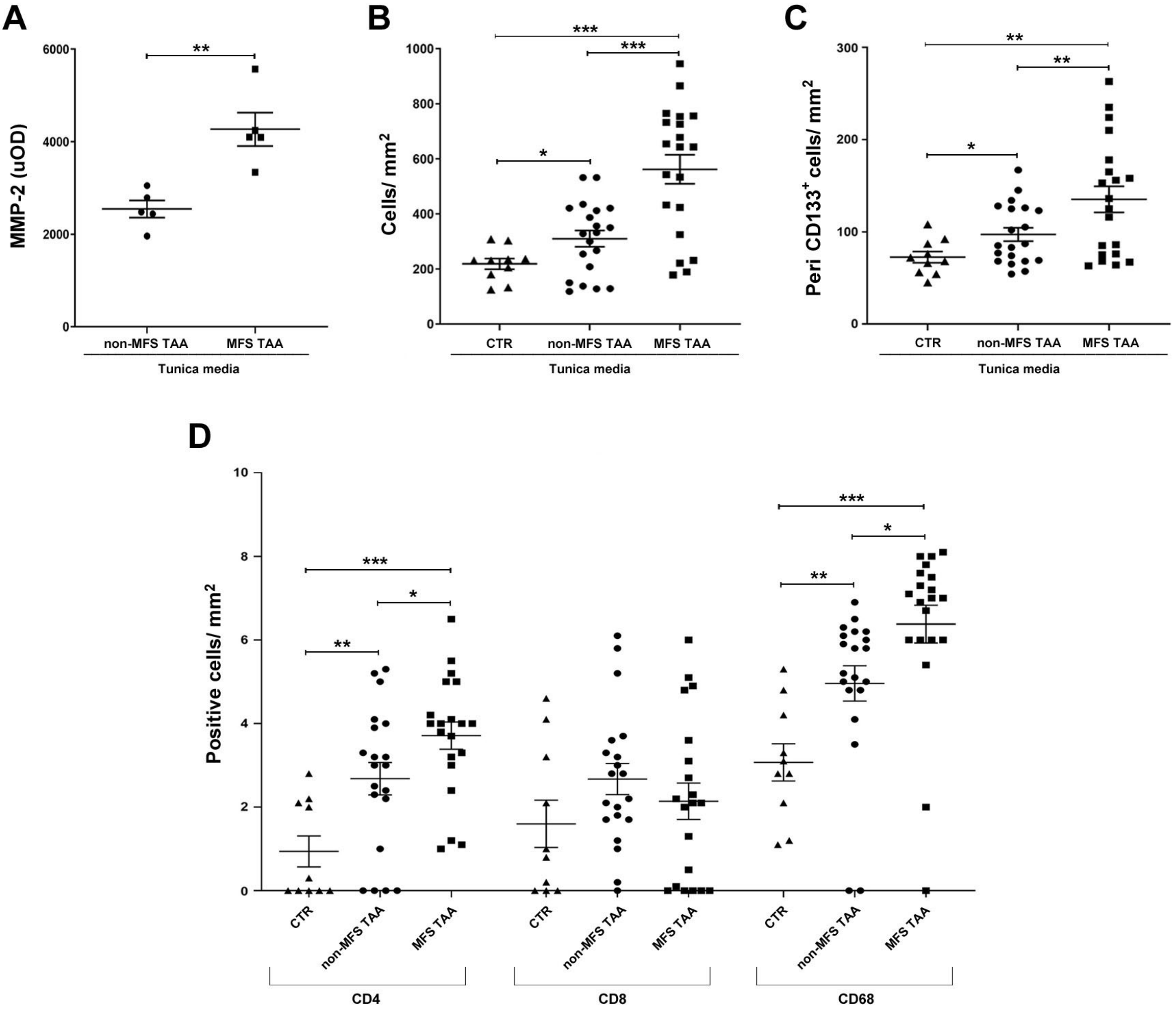

Supplemental Figure III

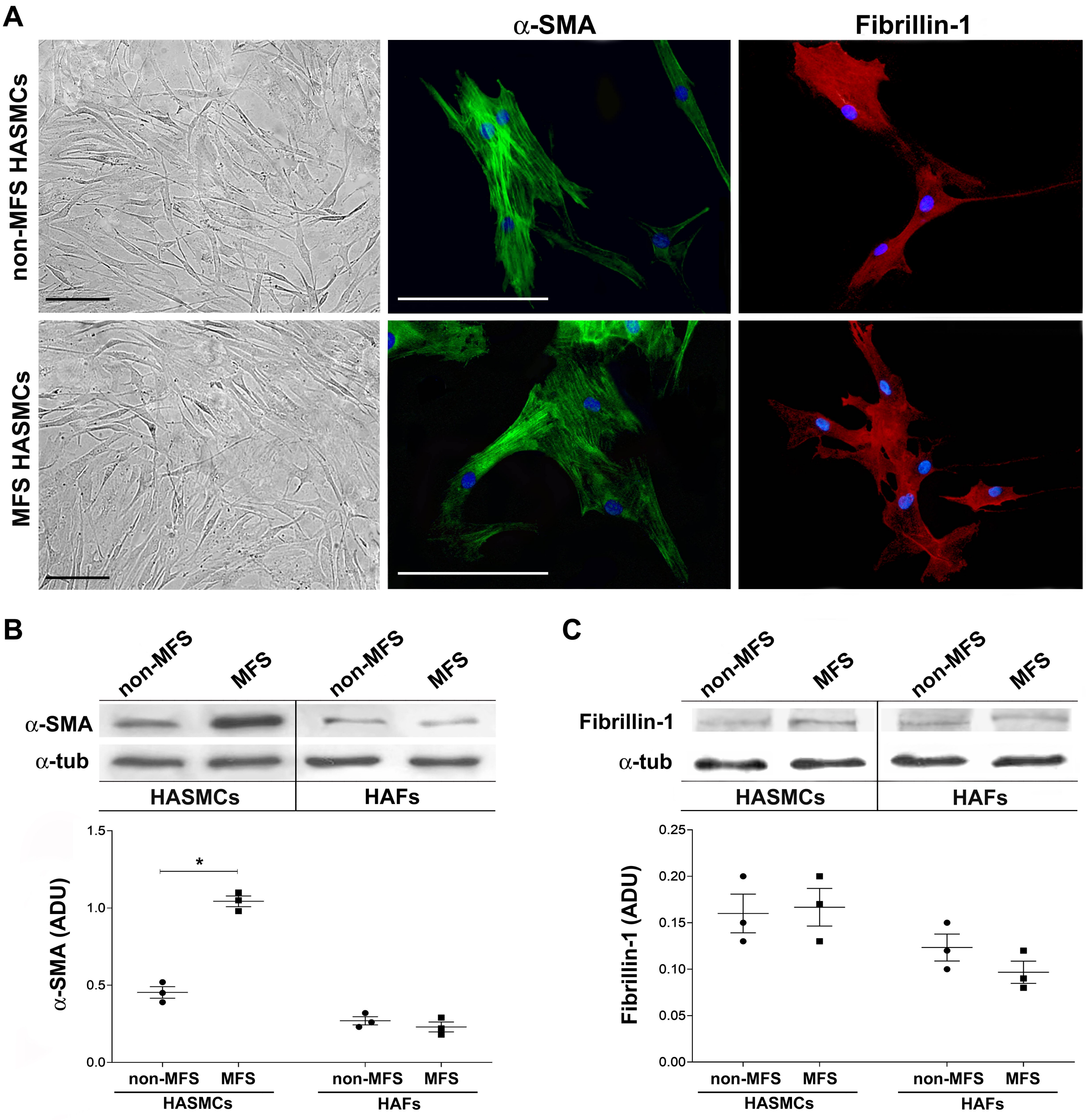

Supplemental Figure IV

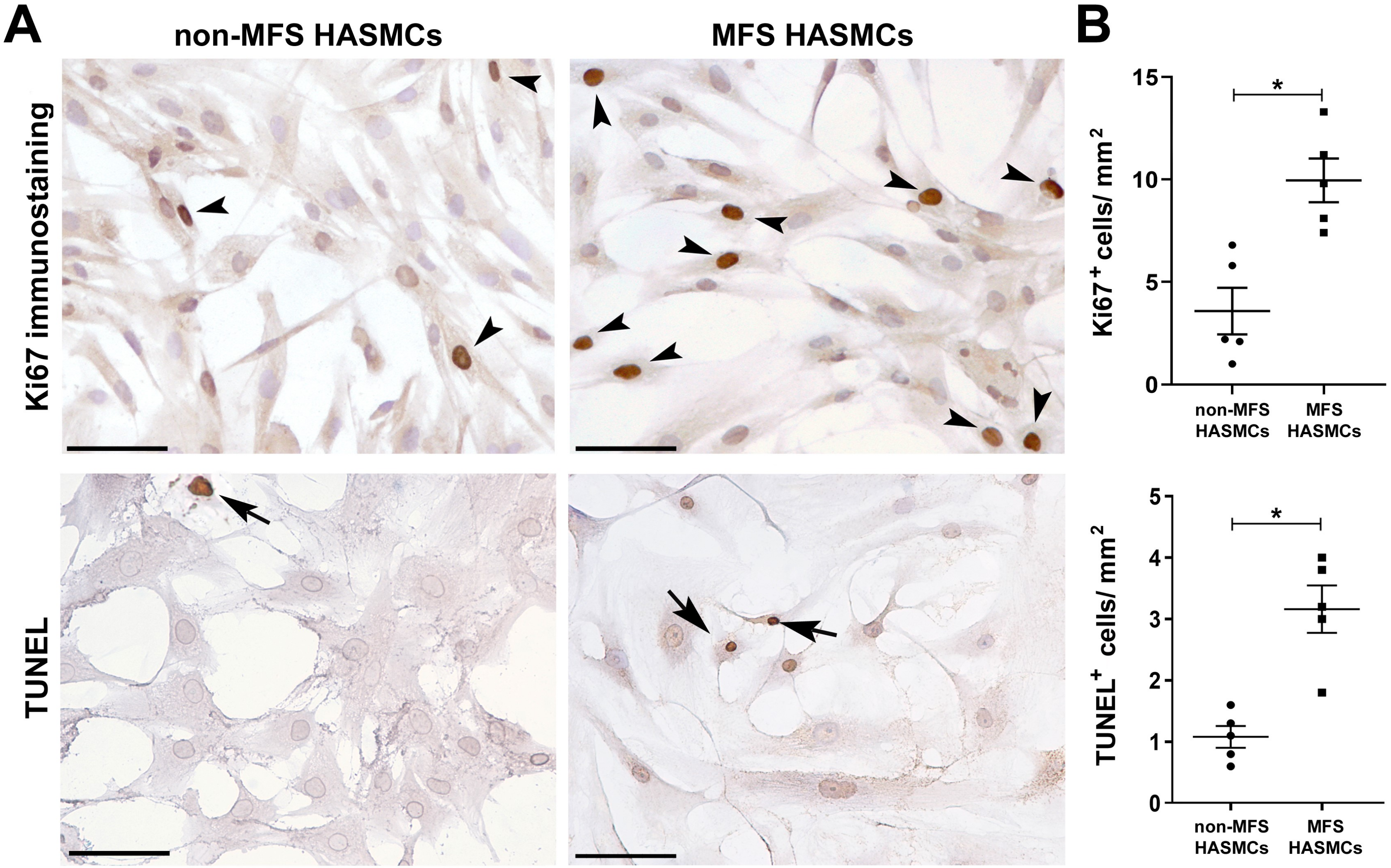

Supplemental Figure V

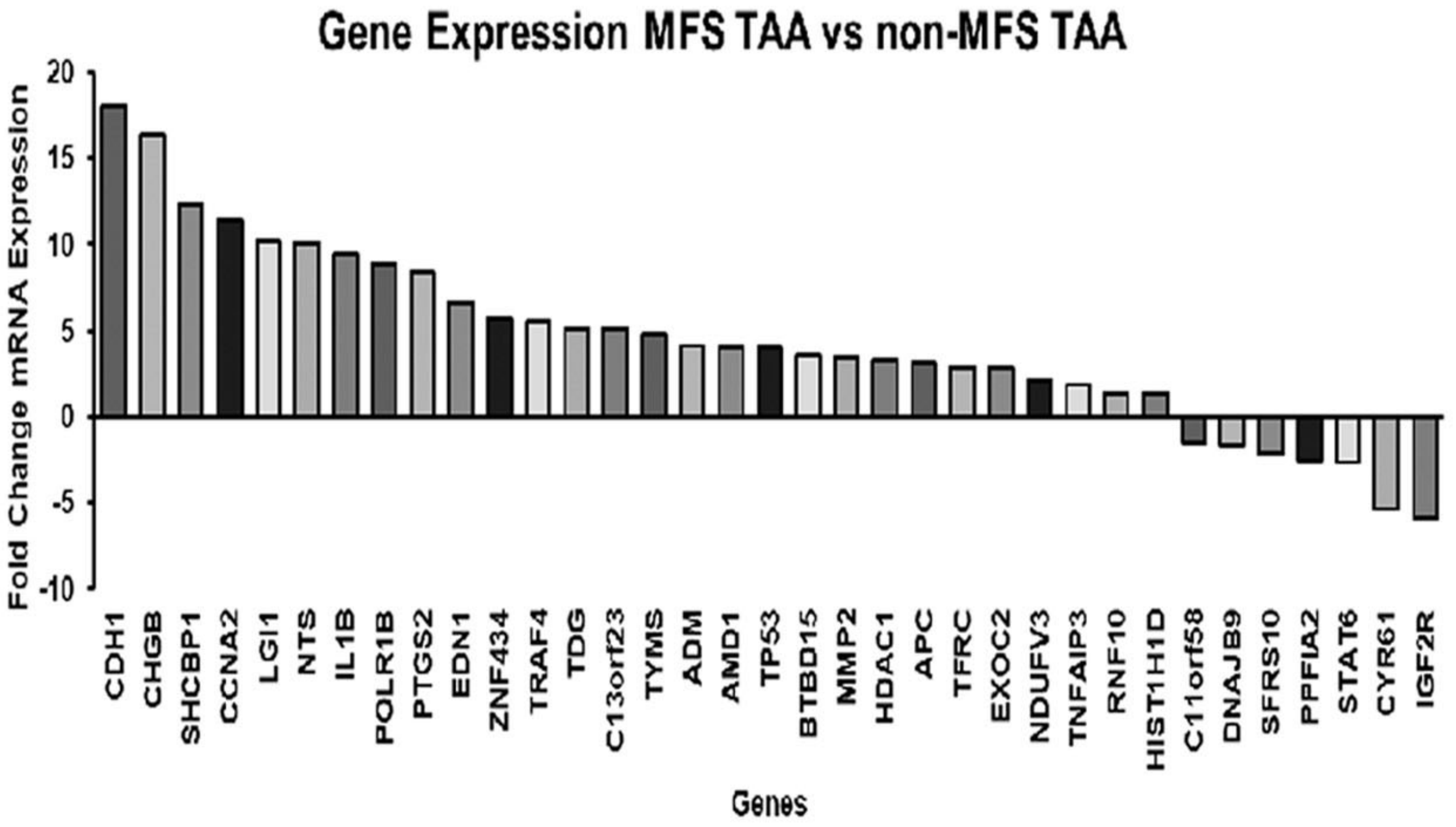

Supplemental Figure VI

Supplemental Figure legend

Supplemental Figure I. Macroscopic, ecocolor Doppler and computed tomography vision of MFS and non-MFS TAA. In the left side, intraoperative view of ascending aortic aneurysm associated to non-MFS and MFS aorta; in the middle, ecocolor Doppler evaluation; in the right side, computed Tomography view of ascending aortic aneurysm. Abbreviations: MFS, Marfan syndrome; non-MFS, non Marfan syndrome; TAA, thoracic aorta aneurysm.

Supplemental Figure II. Histological analysis of control aorta, MFS and non-MFS TAA. Representative aortic media sections stained with Haematoxylin&Eosin (H&E) show accumulation of basophilic material in the intimal and medial tunica of non-MFS and MFS TAA compared with control. Bar graphs of morphometric evaluations concerning intimal and medial thickness. Abbreviations: MFS, Marfan syndrome; non-MFS, non Marfan syndrome; TAA, thoracic aorta aneurysm.

Supplemental Figure III. MMP2 activity, cellularity, vascular precursors and inflammatory recruitment in the tunica media of MFS TAA, non-MFS TAA and control aorta. (*A*) Densitometric analysis in uOD (unit optical density) values of MMP-2 gelatinase activity (N=5/group). *B)* Bar graph shows the cellularity (number of cells/ mm^2) in the tunica media of MFS TAA, non-MFS TAA and control aorta. (*C*) Bar graph shows perivascular $CD133^+$ cell density (number of cells/ mm^2) in the tunica media of MFS TAA, non-MFS TAA and control aorta. (*D*) Semiquantitative evaluation of inflammatory cell infiltrate in outer media and adventitia documents the increase of $CD4^+$ and $CD68^+$. MFS TAA (N=20), non-MFS TAA (N=20) and control aorta (N=10). Average are reported as means ± SEM; **P*< 0.05; ***P*<0.01; ****P*<0.001; estimated by *t*-test. Abbreviations: uOD, unit optical density; MFS, Marfan syndrome; non-MFS, non Marfan syndrome; TAA, thoracic aorta aneurysm.

Supplemental Figure IV. Morphological and phenotypic features of cultured HASMCs from non-MFS and MFS TAA *in vitro*. (A) Phase contrast micrographs at third passage of

cultured HASMCs from non-MFS and MFS TAA; scale bar =150 μm. Immunofluorescence staining shows α-SMA and fibrillin-1 expression in both cultures; scale bar =100 μm. (B) Representatives blots and bar graphs of α-SMA and fibrillin-1 expression in cultured HASMCs and HAFs from non-MFS and MFS TAA (N=3/group). Average are reported as means ± SEM; **P*< 0.001; estimated by *t*-test. Abbreviations: HASMCs, human aortic medial-derived smooth muscle cells; HAFs, human aortic fibroblasts; MFS, Marfan syndrome; non-MFS, non Marfan syndrome; TAA, thoracic aorta aneurysms; α-SMA, α-smooth muscle actin; α-tub, α-tubulin; ADU, arbitrary densitometry units.

Supplemental Figure V. **Increased proliferation and apoptosis characterize cultured HASMCs from MFS TAA.** (*A*) Representative images and (*B*) semiquantitative evaluation of Ki67 immunocytochemistry (dark brown nuclei, arrowheads) and *In situ* end-labeling of DNA fragments by TUNEL assay (dark brown nuclei, arrows) in non-MFS and MFS HASMCs cultures (N=5/group). Scale bar equals to 100μm. Scale bar equals to 50μm. Average are reported as means ± SEM; * *P*<0.01; estimated by *t*-test.

Supplemental Figure VI. Gene deregulation in MFS TAA media tissue. Gene deregulation in MFS TAA expressed as fold change of non-MFS TAA (MFS TAA, N=4; non-MFS TAA, N=2).

Supplemental Table 1. Up and down-regulated miRNAs in MFS TAA.

miRs	Fold Change
hsa-miR-632	13,51
hsa-let-7f-5p	-6,82
hsa-miR-101-3p	-7,47
hsa-miR-125a-3p	-2,91
hsa-miR-1	-3,64
hsa-miR-1537	-8,52
hsa-miR-199b-5p	-75,42
hsa-miR-217	-4,19
hsa-miR-25-3p	-2,76
hsa-miR-27a-3p	-83,49
hsa-miR-27b-3p	-8,03
hsa-miR-28-3p	-2,82
hsa-miR-29c-3p	-18,77
hsa-miR-302d-3p	-2,40
hsa-miR-30d-5p	-33,40
hsa-miR-338-3p	-3,93
hsa-miR-378a-3p+hsa-miR-378i	-14,08
hsa-miR-4461	-24,91
hsa-miR-515-5p	-5,05
hsa-miR-525-3p	-11,36
hsa-miR-592	-9,18
hsa-miR-614	-3,69
hsa-miR-890	-12,61
hsa-miR-26a-5p	-13,07
hsa-miR-29b-3p	-40,96
hsa-miR-145-5p	-3,82

Supplemental Table 2. Up and down-regulated genes in MFS compared to non-MFS TAA.

Upregulated genes		
Gene Name	Accession	Fold Change
CDH1	NM_004360.2	17,98
CHGB	NM_001819.1	16,45
SHCBP1	NM_024745.2	12,38
CCNA2	NM_001237.2	11,44
LGI1	NM_005097.1	10,25
NTS	NM_006183.3	10,17
IL1B	NM_000576.2	9,47
POLR1B	NM_019014.3	8,89
PTGS2	NM_000963.1	8,47
EDN1	NM_001955.2	6,64
ZNF434	NM_017810.2	5,75
TRAF4	NM_004295.2	5,52
TDG	NM_003211.3	5,17
C13orf23	NM_025138.2	5,12
TYMS	NM_001071.1	4,76
ADM	NM_001124.1	4,12
AMD1	NM_001634.4	4,03
TP53	NM_000546.2	4,03
BTBD15	NM_014155.2	3,56
MMP2	NM_004530.2	3,5
HDAC1	NM_004964.2	3,23
APC	NM_000038.3	3,1
TFRC	NM_003234.1	2,89
EXOC2	NM_018303.4	2,82
NDUFV3	NM_021075.3	2,1
TNFAIP3	NM_006290.2	1,87
RNF10	NM_014868.3	1,43
HIST1H1D	NM_005320.2	1,41

Downregulated genes		
Gene Name	Accession	Fold Change
C11orf58	NM_014267.3	-1,54
DNAJB9	NM_012328.1	-1,57
SFRS10	NM_004593.1	-2,21
PPFIA2	NM_003625.2	-2,51
STAT6	NM_003153.3	-2,65
CYR61	NM_001554.3	-5,35
IGF2R	NM_000876.1	-5,97

Supplemental Table 3. Up and down-regulated genes in MFS compared to non-MFS TAA.

Upregulated genes		
**Gene Name**	**Accession**	**Fold Change**
CDH1	NM_004360.2	17,98
CHGB	NM_001819.1	16,45
SHCBP1	NM_024745.2	12,38
CCNA2	NM_001237.2	11,44
LGI1	NM_005097.1	10,25
NTS	NM_006183.3	10,17
IL1B	NM_000576.2	9,47
POLR1B	NM_019014.3	8,89
PTGS2	NM_000963.1	8,47
EDN1	NM_001955.2	6,64
ZNF434	NM_017810.2	5,75
TRAF4	NM_004295.2	5,52
TDG	NM_003211.3	5,17
C13orf23	NM_025138.2	5,12
TYMS	NM_001071.1	4,76
ADM	NM_001124.1	4,12
AMD1	NM_001634.4	4,03
TP53	NM_000546.2	4,03
BTBD15	NM_014155.2	3,56
MMP2	NM_004530.2	3,5
HDAC1	NM_004964.2	3,23
APC	NM_000038.3	3,1
TFRC	NM_003234.1	2,89
EXOC2	NM_018303.4	2,82
NDUFV3	NM_021075.3	2,1
TNFAIP3	NM_006290.2	1,87
RNF10	NM_014868.3	1,43
HIST1H1D	NM_005320.2	1,41

Downregulated genes		
**Gene Name**	**Accession**	**Fold Change**
C11orf58	NM_014267.3	-1,54
DNAJB9	NM_012328.1	-1,57
SFRS10	NM_004593.1	-2,21
PPFIA2	NM_003625.2	-2,51
STAT6	NM_003153.3	-2,65
CYR61	NM_001554.3	-5,35
IGF2R	NM_000876.1	-5,97
